# Meta-Analysis of SNPs Determining Litter Traits in Pigs

**DOI:** 10.3390/genes13101730

**Published:** 2022-09-26

**Authors:** Ewa Sell-Kubiak, Jan Dobrzanski, Martijn F. L. Derks, Marcos S. Lopes, Tomasz Szwaczkowski

**Affiliations:** 1 Department of Genetics and Animal Breeding, Poznań University of Life Sciences, Wołyńska 33, 60-637 Poznań, Poland; 2 Topigs Norsvin Research Centre, Schoenaker 6, 6641 SZ Beuningen, The Netherlands; 3 Topigs Norsvin, R. Visc. do Rio Branco, 1310-Centro, Curitiba 80420-210, PR, Brazil

**Keywords:** corpus luteum number, number of stillborn, gene ontology, genomic regions, gene network, pCADD, protein–protein interaction

## Abstract

Nearly 2000 SNPs associated with pig litter size traits have been reported based on genome-wide association studies (GWASs). The aims of this study were to gather and integrate previously reported associations between SNPs and five litter traits: total number born (TNB), number born alive (NBA), number of stillborn (SB), litter birth weight (LWT), and corpus luteum number (CLN), in order to evaluate their common genetic background and to perform a meta-analysis (MA) of GWASs for total number born (TNB) recorded for animals from five pig populations. In this study, the genes with the largest number of associations with evaluated litter traits were *GABRG3, RBP7, PRKD1,* and *STXBP6*. Only 21 genes out of 233 associated with the evaluated litter traits were reported in more than one population or for more than one trait. Based on this evaluation, the most interesting candidate gene is *PRKD1*, which has an association with SB and TNB traits. Based on GO term analysis, *PRKD1* was shown to be involved in angiogenesis as well. As a result of the MA, two new genomic regions, which have not been previously reported, were found to be associated with the TNB trait. One SNP was located on *Sus scrofa* chromosome (SSC) 14 in the intron of the *FAM13C* gene. The second SNP was located on SSC9 within the intron of the *AGMO* gene. Functional analysis revealed a strong candidate causal gene underlying the QTL on SSC9. The third best hit and the most promising candidate gene for litter size was found within the *SOSTDC1* gene, associated with lower male fertility in rats. We showed that litter traits studied across pig populations have only a few genomic regions in common based on candidate gene comparison. *PRKD1* could be an interesting candidate gene with a wider association with fertility. The MA identified new genomic regions on SSC9 and SSC14 associated with TNB. Further functional analysis indicated the most promising gene was *SOSTDC1*, which was confirmed to affect male fertility in other mammals. This is an important finding, as litter traits are by default linked with females rather than males.

## 1. Introduction

Since the discovery of the first genomic association between litter size and estrogen receptors in 1996 [1], there have been high hopes of finding more major genomic regions for reproduction traits [2]. Currently, one of the most popular methods for uncovering the associations between traits and genes is genome-wide association study (GWAS) using single nucleotide polymorphism (SNP) markers [2,3,4]. Genomic regions related to litter traits were revealed by GWAS in various farm animal species, including rabbits [5,6], goats [7,8], cattle [9], and pigs [10,11,12]. Although GWAS has presented some shortcomings resulting from the rigid structure of the chips and uneven distribution of markers across the chromosomes, those studies still provided the greatest insight into the genetic bases of many reproduction traits in a variety of pig populations [10].

Many results of GWAS and earlier QTL mapping are stored in the Pig QTL database [13]. Although it doesn’t store all associations reported in the literature, this database is a highly important source of information and gathers 30,869 QTLs for 692 traits, with 1625 of those being QTLs for litter traits. Among those QTLs, 356 are related to total number born (TNB), 223 to number born alive (NBA), 137 to number of stillborn (SB), 52 to litter birth weight (LWT), and 130 to corpus luteum number (CLN). The reported QTL regions were in most cases identified using SNPs in GWASs.

Even though those studies were performed on different populations, they often used the same SNP chips and statistical methodology. However, the results of those studies are not repeatable across breeds or even within one breed. This lack of overlap among GWAS results for TNB and NBA was recently described in a review by Bakoev et al. [12]. Moreover, litter traits proved to be highly complex polygenic traits. This, in contrast to production traits such as backfat and growth rate that have several major genes confirmed [14,15,16], resulted in detection of only one major QTL, the previously mentioned estrogen receptor [1].

Despite the high number of studies and QTLs reported for litter traits, no research aimed at reviewing the existing results of GWASs for several litter traits in a more systematic manner. One of the main approaches to integrate and pool the results from single GWASs is a meta-analysis (MA). The GWAS MA is widely used in medical research and is becoming more popular in livestock studies [17,18,19]. Meta-analysis could also be used to increase the power of association studies by combining datasets from different sources and reducing false positive associations [20,21]. This also could help indicate new major QTLs for litter traits.

Therefore, the aims of this study were to (1) gather and integrate previously reported associations between SNPs and five litter traits: total number born (TNB), number born alive (NBA), number of stillborn (SB), litter birth weight (LWT), and corpus luteum number (CLN) to evaluate their common genetic background; (2) investigate the relationships among reported candidate genes for litter traits by searching for functional interactions among proteins encoded by those genes; and (3) combine the full GWAS results from several studies to identify the effects of gene sets in a meta-analysis.

## 2. Materials and Methods

This study’s main objectives were to: (1) gather and integrate previously reported associations between SNPs and five litter traits: total number born (TNB), number born alive (NBA), number of stillborn (SB), litter birth weight (LWT), and corpus luteum number (CLN) to evaluate their common genetic background; (2) investigate the relationships among reported candidate genes for litter traits by searching for functional interactions among proteins encoded by those genes; and (3) combine the full GWAS results from several studies to identify the effects of gene sets in a meta-analysis. This was performed using two datasets. Dataset **A** (Tables S1–S5; Supplementary_Tables) refers to the data generated from significant SNPs reported in studies performed for five traits: total number born (TNB), number born alive (NBA), number of stillborn (SB), litter birth weight (LWT), and corpus luteum number (CLN). Dataset **B** refers to data created from full GWAS results for TNB generated in five studies: [10,22,23,24,25] (Table 1).

### 2.1. Dataset A

To create dataset **A**, this part of the study was performed following the guidelines from “Genome-wide association studies meta-analysis” by Thompson et al. [26]. The selection of published GWAS results included in the analysis was conducted from March 2020 to December 2020 within the following databases: Web of Knowledge, Web of Science, PubMed, and Google Scholar. Studies reporting associations between SNPs and TNB, NBA, SB, LWT, or CLN were collected using various combinations of the following terms: “pig”, “litter size”, “total number born”, “number born alive”, “litter size”, “SNP”, “litter birth weight”, ”stillbirth”, “born dead”, “ovulation”, “corpus luteum number”, “polymorphism”, and “GWAS”. Later, the survey was supported by searching for QTL associations in the Pig QTL database and also by screening the references of retrieved papers. The complete data collected for dataset **A** are presented in Supplementary_Tables in Tables S1–S5, which include *Sus scrofa* chromosome (SSC) location, allele, candidate gene, and breed. The general overview of dataset **A** is presented in Table 2.

### 2.2. Dataset B

Dataset **B** was created by merging complete results of GWAS for TNB from five publications: Uimari et al. [22], Sell-Kubiak et al. [23], Ma et al. [25], Balogh et al. [24], and Zhang et al. [10]. The dataset created after merging the results from the five GWASs consisted of 63,531 SNPs available for further analysis. A specific overview of data collected in dataset **B** is presented in Table 1. In short, the data came from five different populations: Finnish Landrace, Large White, Erhualian, Duroc, and Hungarian Large White, and different methods were applied to perform the GWASs: single-SNP mixed model with pedigree additive relationship matrix or with genomic relationship matrix, and multiple-SNP Bayesian approach (Table 1). All populations were genotyped with Porcine SNP60 Bead Chip. The GWAS data from Sell-Kubiak et al. [23] covered phenotypes presented in a published paper, whereas the *p*-values of SNPs are the result of the single-SNP GWAS with genomics relationship matrix (more details in Supplementary_Method) and were not published in the mentioned paper.

### 2.3. Gene Ontology Analysis

Analysis of gene ontology (GO) terms [45] assigned to candidate genes collected in dataset **A** was performed using the PANTHER classification system Version 16 [46] with the newest available Ensembl Sscrofa11.1 as a reference genome. The analysis was performed with the binomial test of overrepresentation to explore biological processes in which the candidate genes are involved [46]. Results from the PANTHER were considered statistically significant at a false discovery rate (FDR)-corrected *p*-value ≤ 0.05 [47]. This analysis was performed on all candidate genes for five litter traits simultaneously.

### 2.4. Gene Network Analysis

The gene network analysis was performed with GeneMANIA [48] as a plug-in to Cytoscape v3.8.2 [49], with the human gene annotation as a reference. Settings allowed the authors to evaluate co-expression, physical interaction, gene interactions, shared protein domains, and co-localization. This analysis was performed only on candidate genes from dataset **A** that either had at least two SNPs detected along their sequence (Table 3) or were associated with more than one trait or presented in more than one study (Table 4).

### 2.5. Protein–Protein Interactions

Interactions between proteins coded by candidate genes reported in dataset **A** were investigated using a protein–protein interactions network using STRING Genomics v.11 [50]. The level of confidence for observed protein–protein interactions was limited to medium confidence interactions with scores > 0.40 with remaining settings at default [51].

### 2.6. Meta-Analysis on Dataset B

To combine estimates of SNP associations for TNB obtained from different populations, MA was performed on dataset **B**. Based on Garrick et al. [52], the decision was made that despite different definitions of phenotypes for TNB, the five datasets can be combined into one. All available 63,531 SNPs were included in the MA based on the weighted Z-score model. This approach considers the *p*-value, direction of effect, and number of individuals present in each study and was performed using METAL software [53]. The weighted Z-score model was chosen in accordance with Van den Berg et al. [54], who indicated this method as the most preferable when combining GWAS results with differences in the definition of the phenotypes, as is present in dataset **B**. Post-MA, the Bonferroni correction was applied to establish statistically significant associations. In addition, MA was performed in several runs with subsets of SNPs as follows: with all SNPs, with SNPs from at least 2 populations, with SNPs from at least 4 populations, and with SNPs present in all populations.

The evaluation of candidate genes found with the MA was performed with Bgee Version 14.2 (https://bgee.org/api/, accessed on 26 August 2022), GeneCards [55] and Ensembl BioMart.

### 2.7. Candidate Genes and Causal Variants

To assess possible candidate genes and variants underlying GWAS peaks, we also used the pCADD pipeline as described in [56]. In short, we extracted all sequence variants in linkage disequilibrium (LD) with the top SNP from the meta-GWAS. The candidate variants in LD with the top SNP were ranked according to their pCADD score. pCADD provides a per-base impact score [57] to distinguish between variants that likely have impact (positive or negative) and variants that are benign. The pipeline additionally provides gene annotation and functional genomic information to further fine-map the QTL region.

## 3. Results

In this study, two datasets were analyzed. First, dataset **A** (Tables S1–S5; Supplementary_Tables) refers to the data generated from significant SNPs reported in GWASs performed for five traits: total number born (TNB), number born alive (NBA), number of stillborn (SB), litter birth weight (LWT), and corpus luteum number (CLN). Second, dataset **B** refers to data created from full GWAS results for TNB generated in five studies [10,22,23,24,25] (Table 1).

### 3.1. SNPs and Candidate Genes from Dataset A

In total, 24 papers that studied at least one of the litter traits of interest were found (Tables S1–S5; Supplementary_Tables). Most of these studies focused on TNB and NBA, while studies for SB, LWT, and CLN were more limited (Table 2). The most common breeds evaluated in these studies were Large White, Yorkshire, Landrace, Duroc, and their crosses, with occasional occurrence of Erhualian and Berkshire. The definition of phenotypes varied across the publications and traits. While most of the publications used direct measurement of the trait, some authors chose deregressed breeding values as phenotypes [10,22,23]. In addition, in two studies, TNB phenotypes were divided into TNB at the first parity and the later parities [22,25].

The highest number of associations among SNPs and analyzed traits was reported as expected for TNB and NBA (Table 2), as those were the most-studied traits. Identified SNPs were rarely placed within the candidate gene. Most SNPs identified to be associated with the evaluated traits were located at least 50 Kbp away from the candidate gene. Only in the case of TNB, NBA, and SB were at least two SNPs with significant association with these traits in one population located within the candidate gene regions (Table 3). Genes with the largest number of associations along their sequences were *GABRG3, RBP7, PRKD1,* and *STXBP6*. Furthermore, only 21 genes out of 233 associated with the selected litter traits were reported in more than one population or for more than one trait (Table 4). For one gene out of this list (*DDAH1)* the overlap was expected, as the two studies reporting it were based on data from the same population [25,29]. This was not the case for the remaining populations presented in Table 4.

### 3.2. GO Term Analysis Results

In total 34 GO terms related to the biological processes were found (FDR < 0.05; Table 5). The most promising biological processes were “positive regulation of blood vessel endothelial cell migration”, describing genes *NRP1, PIK3C2A, HDAC9, AKT3,* and *PRKD*, and “positive regulation of cell migration involved in sprouting angiogenesis”, describing *NRP1, PIK3C2A, HDAC9,* and *AKT3*. Surprisingly, the majority of the remaining GO terms were involved with processes in nervous system development or regulation.

### 3.3. Gene Network and Protein–Protein Interactions

**Figure 1:**
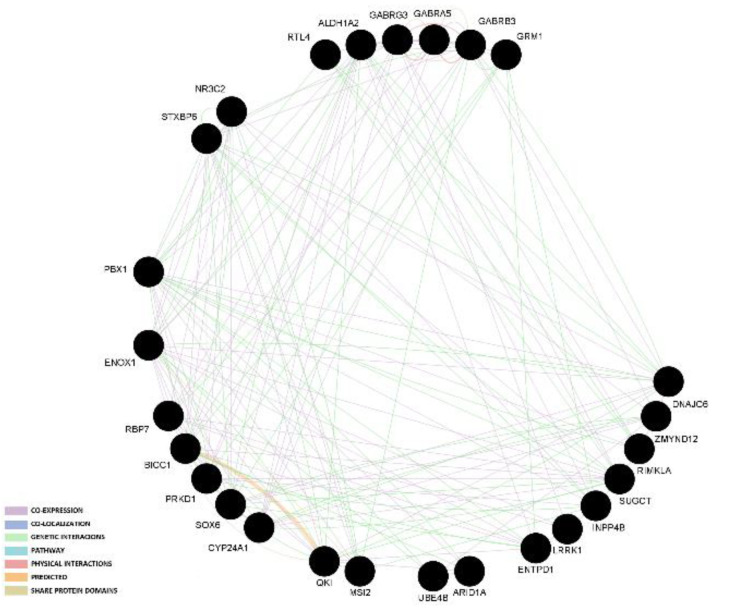
Gene network analysis of candidate genes that had at least two SNPs associated with reproduction traits.

**Figure 2:**
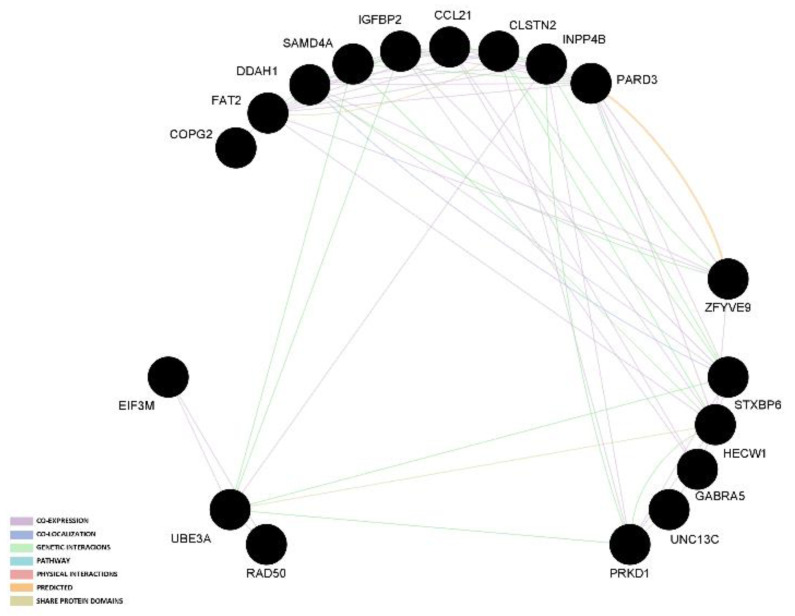
Gene network analysis of candidate genes that had an association reported with at least two reproduction traits or in two different populations.

The two gene network analyses indicated that most links among genes are based on co-expression and genetic interaction. The graphical representation of those results in presented in Figure 1 and Figure 2.

**Figure 3:**
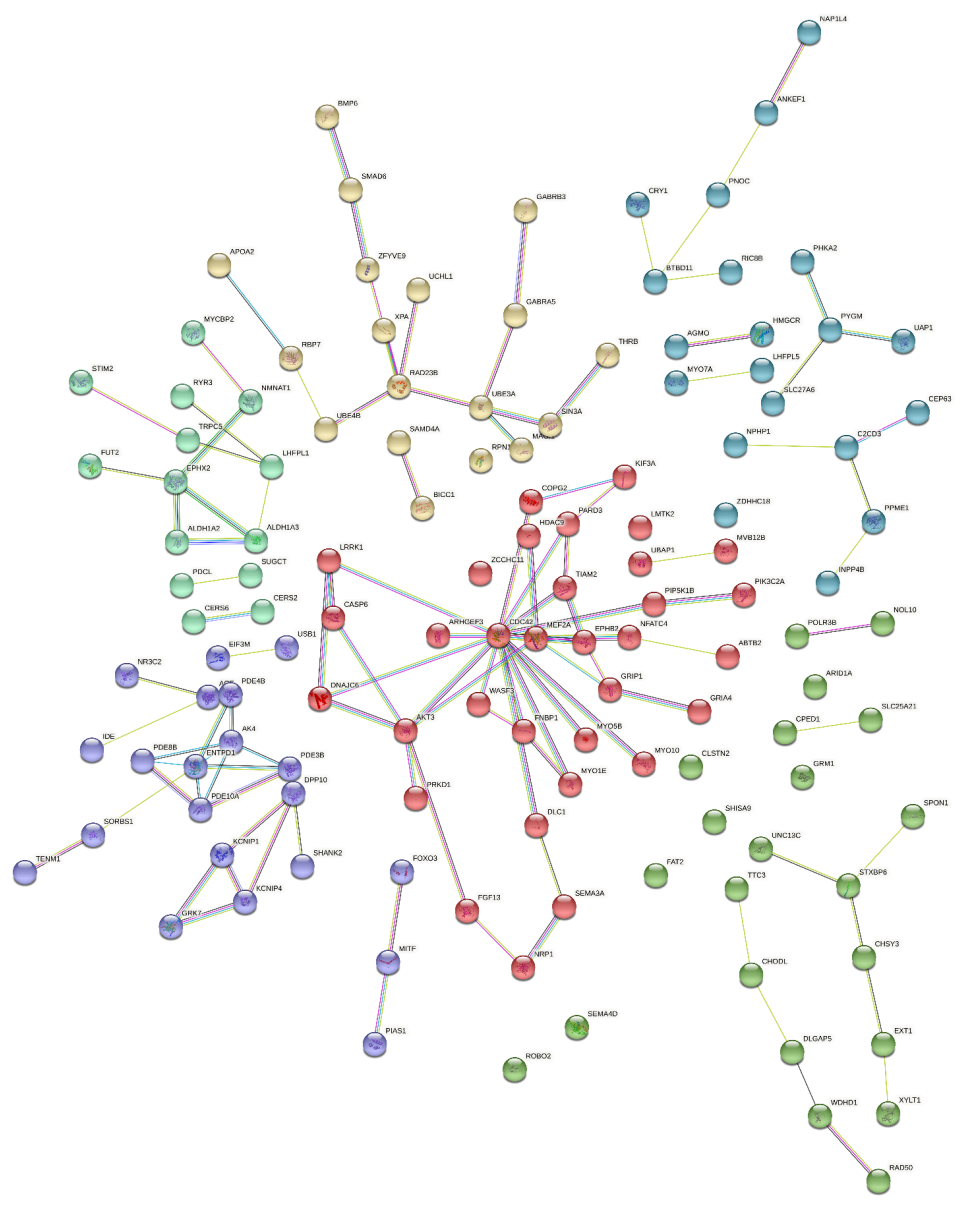
Protein–protein interaction network analysis of genes associated with total number born, number born alive, number of stillborn, litter birth weight, and corpus luteum number in pigs. Types of interactions: teal line—known interaction based on curated database, pink line—known interaction experimentally determined, green line—predicted interaction based on gene neighborhood, red line—predicted interaction based on gene fusion, blue line—predicted interaction based on a gene co-occurrence.

Interactions among proteins (protein–protein interaction network; PPIN) coded by genes associated with the analyzed traits are presented in Figure 3. In total, 174 genes formed some pairs or chains of interactions with one evident cluster built of 138 proteins and 12 short chains that contained a maximum of 7 genes coding those proteins. The genes with the largest number of proven links among coded proteins were *CDC42*, *LRRK1,* and *AKT3*.

Overall, those two analyses did not provide strong evidence for interactions among candidate genes for the litter traits.

### 3.4. Meta-Analysis of Five GWA Studies

As a result of the MA performed on dataset B, two SNPs were found to be significantly associated with TNB. The first, rs80945731, located on SSC14: 62633073, was available in four out of five evaluated populations [10,22,24,25]. Based on Ensembl Sscrofa11.1, this SNP is located in the intron region of the *FAM13C* gene. The second, rs81300422, located on SSC9: 84696142, was unfortunately present in only one population [22]. According to Ensembl Sscrofa11.1, this gene is located within the intron of the gene *AGMO*. Importantly, these two SNPs found in the MA are reported for the first time to have an association with TNB. The last run of MA, which included only SNPs present in all populations, did not show any significant association with the evaluated traits.

### 3.5. Candidate Genes and Causal Variation

To assess candidate causal genes at the two QTL loci, we ran the pCADD-GWAS pipeline in four different breeds from Topigs Norsvin [45]. The rs81300422 SNP, which is located on SSC9, was segregated only in a synthetic boar line with a 7% minor allele frequency. The third best hit with the pCADD pipeline (after two intergenic SNPs) is upstream of the *SOSTDC1* gene. The *SOSTDC1* gene is a member of the sclerostin family functioning as a bone morphogenetic protein (BMP) antagonist, which is known to be associated with fertility. The descriptive statistics for TNB, NBA, SB, and mummies per genotype of rs81300422 is presented in Table 6. It can be observed that animals being homozygous for the alternative allele tend to lower SB (in sows and boars from a synthetic boar line and its crossbreeds) and higher rate of mummies (in sows from a synthetic boar line and in crossbreed sows) than homozygous animals for the reference allele. This is, however, not a statistically significant difference.

The rs80945731 SNP on SSC14 was common in all four commercial breeding populations. The results yielded SNPs within and close to the *PHYHIPL* and *FAM13C* genes, thus partially overlapping with the results of the candidate gene search applying the classical approach.

## 4. Discussion

This study intended to evaluate existing knowledge about the genomic regions associated with five litter traits in pigs: total number born (TNB), number born alive (NBA), number of stillborn (SB), litter birth weight (LWT), and corpus luteum number (CLN), and search for new candidate genes using bioinformatics analysis on combined results from previous studies. This study is the first one to evaluate the possibility of a common genetic background for those litter traits, as well as to present a meta-analysis for SNPs associated with TNB and combining data from five different populations.

### 4.1. Genetic Relationship between Litter Traits

One of the hypotheses at the beginning of this study was that there must be overlapping genomic regions and candidate genes among the evaluated litter traits. This hypothesis was based mostly on strong positive genetic correlations among TNB, NBA, CLN, and LWT [58,59]; however, it was not confirmed with the data collected in dataset **A**. Only one candidate gene, *ZFYVE9*, was reported for three litter traits (TNB, NBA, and CLN) and in two populations [31,42]. Another three candidate genes for SB (with a negative genetic correlation with the remaining litter traits [59]) were also found to be associated with TNB (*STXBP6* [11,29]), NBA (*PRKD1* [11,28]) or CLN (*SAMD4A* [42,43]). For LWT, only one candidate gene was in common with TNB (*COPG2* [28]), whereas only one candidate gene for SB overlapped between two populations (*MAPK1P1L* [43,44]). The abovementioned relationships among traits could suggest some pleiotropic effect of those genes on litter traits. In two populations three candidate genes for TNB were also reported: *ASIC2* [29,37], *GABRA5* [28,37], and *NEK10* [29,37]. Even though some connections across studies reporting candidate genes for litter traits can be observed, considering that we studied 233 candidate genes in total and only 21 of them covered more than one trait or population, one cannot assume the common genetic background of those traits. However, for production traits in pigs, except for major genes, such overlap between traits or populations is also not common [13]. It should be mentioned here that the older studies from PigQTLdb [13] reported very long QTLs because of high LD or access to SNP chips with low density. Thus, some of the reported candidate genes might in fact be very distant from the actual SNPs associated with the litter traits. Another reason might be the population stratification, which if not accounted for, can lead to false positives (e.g., [23]).

The lack of overlap between traits in terms of candidate genes was present not only among the studies for one trait but also within the same study if it focused on two or more traits. Moreover, more than one SNP was rarely reported within the candidate gene in the same population. Thus, the lack of overlap was not caused by the differences among the populations or the methodologies behind detecting associations, which are mentioned as two main reasons for differences among compared GWASs [12]. Even more so, the low repeatability of results across populations is surprising, because most of the studies were based on the same SNP chip and (in general) the most popular pig breeds. Another reason might have been the low heritability of the studied traits, which based on different studies varies from 0.05 to 0.2 [11,28,59,60]. The heritability level affects the ability to retrace the trait’s heritability based on SNP associations [23].

Those results clearly show that the polygenic characteristics of the litter traits are very complex, and despite the undeniable relationship among traits their genetic background is mostly affected by different genomic regions often involved in nervous system development.

### 4.2. Connections among Candidate Genes

The gene network and protein–protein interaction analysis did not indicate clear clusters among candidate genes. It needs to be noted, however, that those analyses were based only on linking selected candidate genes with existing databases. This is why the current study did not produce gene expression or any other empirical data that could have been included as additional information for candidate genes. In addition, pig genomic databases are lacking information in comparison with human, mouse, or even cattle gene databases. Thus, the majority of the evidence suggesting functional links among analyzed genes were based on the co-expression of putative homologs present in other organisms (Ensembl Sscrofa11.1). In only a few cases were links between proteins experimentally determined or proteins involved in the same pathway [55].

Some similarities among litter traits can be seen in the GO terms analysis performed on dataset **A**. This analysis clearly showed that genes involved in neurodevelopment and the nervous system are overrepresented among candidate genes for the studied litter traits. This came as a surprising result as we expected to see more genes involved in vasculogenesis and angiogenesis, as blood supply to the uterus and developing fetus was indicated in the past as one of the most limiting factors for litter size [22,61]. That is why from all the possible biological processes that were significant in GO term analysis, we are certain that those involved in positive regulation of blood vessel endothelial cell migration and positive regulation of cell migration involved in sprouting angiogenesis require the most attention.

Interestingly, out of the five genes that have the aforementioned GO terms annotated to them, *PRKD1* is the gene with five SNPs along its sequence associated with number of stillborn [11] and one SNP with TNB [29]. *PRKD1* encodes a protein kinase involved in many cellular processes, including cell migration and differentiation, cell survival, and regulation of cell shape and adhesion [55]. Mutation in this gene causes congenital heart defects in humans [62,63]. In addition, it was also indicated as a candidate gene for age at puberty in maternal and terminal Landrace, Duroc, and Yorkshire lines [64]. Thus, it is associated with at least three traits related to reproduction in their different populations. This indicates that *PRKD1* should be one of the genes considered for molecular analysis involving gene expression or sequencing to confirm its association with litter traits in pigs.

### 4.3. The New Genomic Region Associated with Litter Size

The meta-analysis of GWAS results coming from five different populations indicated two new SNPs associated with litter size. Even though our MA produced far fewer significant results than each of the GWASs separately, this is the first time those SNPs are reported as associated with litter size.

The first of the significant SNPs on SSC9, rs81300422 (genotyped only in one population) is located in the intron of the gene *AGMO* encoding enzyme Alkylglycerol monooxygenase, not very well studied in swine, which is involved in the degradation of ether lipids [65] and the only enzyme that can breakdown alkylglycerols and lysol alkyl glycerophospholipids [66]. This gene was shown to be associated with neurodevelopmental disorders in humans [67], e.g., autism [68], type 2 diabetes [69,70,71], and immune defense [66]. In humans, this gene was expressed mostly in tissues of the digestive tract, especially in the liver, but also in the ovaries, uterus, prostate, and testes [55]. However, the literature does not report any direct links of *AGMO* with fertility.

Thus, a far more interesting and promising candidate gene is *SOSTDC1*, indicated by pCADD analysis, which has been found to affect fertility in male rats [72]. *SOSTDC1* is a negative regulator of spermatogenesis, and downregulation of this gene during puberty is essential for quantitatively and qualitatively normal spermatogenesis governing male fertility. The association between TNB and male fertility comes as a surprise, as litter traits are in general linked with females. However, in some pig breeds it is observed that male genetics plays a more important role in final litter size than originally expected [73,74]. Further comparison of litter traits among pigs with different genotypes for rs81300422 showed that the detected SNP could be affecting the number of stillborn and mummies in pig populations available for pCADD. This was, unfortunately, not confirmed by the statistical analysis.

The second significant SNP, rs80945731 (present in four populations), is located within the gene *FAM13C*, which does not have a clear function in pigs. Nonetheless, it was expressed in swine adipose tissue and tissues of the nervous system (e.g., amygdala or medulla oblongata). At the same time, in humans *FAM13C* was shown to be associated with endometrial mixed adenocarcinoma [55] as well as being used as a marker for prostate cancer [75] or as a rectal adenocarcinoma survival predictor [76]. In humans, this gene also was expressed (among others) in the tissue of the ovaries and uterus as well as the prostate and testes [55]. The second gene indicated by pCADD for rs80945731 was *PHYHIPL*, which is differentially expressed among early, full, and expanded blastocysts in bovine IVP embryos [77]. Both *FAM13C* and *PHYHIPL* in humans were expressed (among others) in the tissue of the ovaries and uterus as well as the prostate and testes [55].

## 5. Conclusions

We have shown that litter traits (total number born, number born alive, number of stillborn, litter birth weight, and corpus luteum number) studied across pig populations have only a few genomic regions in common based on candidate gene comparison. The most interesting candidate gene is *PRKD1*, which has an association with SB and TNB as well as being involved in angiogenesis based on GO term analysis. Our meta-analysis of GWAS results coming from five populations helped identify the new genomic regions on SSC9 and SSC14 associated with TNB. Further pCADD analysis indicated the most promising gene was *SOSTDC1*, which actually has a confirmed effect on male fertility. This is an important finding, as litter traits are by default linked with females rather than males.

## Figures and Tables

**Table 1:** Overview of phenotypic and genomic data available for meta-analysis of GWAS results for total number born (TNB) collected in dataset **B**.

Source of Data	Uimari et al. [22]	Sell-Kubiak et al. [23]	Ma et al. [25]	Zhang et al. [10]	Balogh et al. [24]
Population	Finnish Landrace	Large White	Erhualian	Duroc	Hungarian Large White
Phenotype	deEBV ^1^	deEBV ^1^	EBV ^2^	deEBV ^1^	TNB
BeadChip	Porcine SNP60 Bead Chip				
Individuals	328	2351	48	1067	290
Available SNPs	57,868	40,969	28,020	32,147	56,592
Method	single-SNP mixed model with pedigree relationship matrix	single-SNP mixed model with genomic relationship matrix	single-SNP mixed model with pedigree relationship matrix	single-SNP mixed model with genomic relationship matrix	multi-SNP mixed model with genomic relationship matrix
Detected SNPs	5	0	5	7	3
Threshold	−log_10_(*p*-value) ≤ 5.7 ^3^	−log_10_(*p*-value) ≥ 5	−log_10_(*p*-value) ≤ 5.75 ^3^	−log_10_(*p*-value) ≥ 4	−log_10_(*p*-value) ≥ 5

^1^ deEBV, i.e., deregressed breeding values of total number born; used to remove parents’ average. ^2^ EBV, i.e., direct breeding values of total number born. ^3^ Bonferroni corrected *p*-value.

**Table 2:** Publications reporting SNPs and genes associated with a total number of piglets born (TNB), number born alive (NBA), number of stillborn (SB), or litter birth weight (LWT).

Trait	Number of SNPs	Number of Genes	Publication	
TNB	1	1	An et al.	[27]
	13	7	Coster et al.	[28]
	145	83	He et al.	[29]
	3	0	Kumchoo and Mekchay	[30]
	1	1	Li et al.	[31]
	1	1	Liu et al.	[32]
	7	5	Ma et al.	[25]
	4	4	Sato et al.	[33]
	10	7	Sell-Kubiak et al.	[23]
	5	2	Uimari et al.	[22]
	2	2	Uzzaman et al.	[34]
	10	5	H. Wang et al.	[35]
	1	1	Y. Wang et al.	[36]
	40	6	Wu et al.	[37]
	5	3	Wu et al.	[38]
	7	4	Zhang et al.	[10]
NBA	1	1	An et al.	[27]
	17	8	Bergfelder-Drüing et al.	[39]
	11	6	Chen et al.	[11]
	3	3	Coster et al.	[28]
	3	3	Kumchoo and Mekchay	[30]
	1	1	Li et al.	[31]
	9	7	Ma et al.	[25]
	2	2	Sato et al.	[33]
	27	11	Suwannasing et al.	[40]
	1	0	Uzzaman et al.	[34]
	5	3	Y. Wang et al.	[36]
	101	19	Wu et al.	[37]
	15	6	Wu et al.	[38]
SB	46	22	Chen et al.	[11]
	6	3	Onteru et al.	[41]
	13	11	Schneider et al.	[42]
	2	1	Uimari et al.	[22]
	22	15	Verardo et al.	[43]
LWT	1	1	Coster et al.	[28]
	1	1	Liu et al.	[32]
	10	2	Zhang et al.	[10]
CLN	24	12	Schneider et al.	[44]

**Table 3:** Genes with at least two SNPs in their sequence associated with total number born (TNB), number born alive (NBA), or number of stillborn (SB).

Trait	Gene	Number of SNPs	Publication	
TNB	*GABRG3*	5	Coster et al.	[28]
	*MSI2*	3	He et al.	[29]
	*BICC1*	2	Zhang et al.	[26]
	*ENTPD1*	2	He et al.	[29]
	*ENOX1*	2	Sell-Kubiak et al.	[23]
	*SUGCT*	2	Sell-Kubiak et al.	[23]
	*NR3C2*	2	Wu et al.	[38]
	*GABRB3*	2	Coster et al.	[28]
NBA	*RBP7*	5	Suwannasing et al.	[40]
	*ARID1A*	3	Chen et al.	[11]
	*LRRK1*	2	Suwannasing et al.	[40]
	*ZMYND12*	2	Suwannasing et al.	[40]
	*RIMKLA*	2	Suwannasing et al.	[40]
	*RTL4*	2	Suwannasing et al.	[40]
	*UBE4B*	3	Suwannasing et al.	[40]
	*ALDH1A2*	2	Wu et al.	[37]
	*GABRA5*	2	Wu et al.	[37]
	*INPP4B*	2	Wu et al.	[37]
	*DNAJC6*	2	Wu et al.	[37]
	*QKI*	2	Wu et al.	[37]
	*SOX6*	2	Wu et al.	[37]
SB	*PRKD1*	5	Chen et al.	[11]
	*STXBP6*	4	Chen et al.	[11]
	*PBX1*	2	Chen et al.	[11]
	*GRM1*	2	Chen et al.	[11]
	*CYP24A1*	2	Verardo et al.	[43]

**Table 4:** Genes with at least two SNPs in their sequence associated with more than one of the reproduction traits: total number born (TNB), number born alive (NBA), number of stillborn (SB), or litter birth weight (LWT), or reported in more than one population.

Candidate Gene	Publication and Population											
	An et al. [27]	Chen et al. [11]	Coster et al. [28]	He et al. [29]	Li et al. [31]	Ma et al. [25]	Sato et al. [33]	Schneider et al. [44]	Schneider et al. [42]	Verardo et al. [43]	Wu et al. [37]	Y. Wang et al. [36]
	Berkshire	Duroc	Large White	Erhualian	Yorkshire	Erhualian	Large White	Landrace x Duroc	Duroc, Yorkshire, Landrace	Large White	Yorkshire	Large White
*ASIC2*				TNB							TNB	
*CCL21*											TNB, NBA	
*CLSTN2*				TNB, NBA								
*COPG2*			TNB, LBW									
*DDAH1*				TNB		TNB, NBA						
*EIF3M*			TNB, NBA									
*FAT2*											TNB, NBA	
*GABRA5*			TNB								TNB	
*HECW1*												TNB, NBA
*IGFBP2*	TNB											
*INPP4B*											TNB, NBA	
*MAPK1IP1L*									SB	SB		
*NEK10*				TNB							TNB	
*PARD3*						TNB, NBA						
*PRKD1*		SB	NBA									
*RAD50*							TNB, NBA					
*SAMD4A*								CLN		SB		
*STXBP6*		SB		TNB								
*UBE3A*			TNB, NBA									
*UNC13C*											TNB, NBA	
*ZFYVE9*					TNB, NBA			CLN				

**Table 5:** Gene Ontology analysis for genes associated with total number born, number born alive, number of stillborn, litter birth weight, and corpus luteum number in pigs.

GO Term	Biological Processes (with Hierarchy When Applicable)					Observed Genes	Expected Genes	FDR
9987	cellular process					177	148.91	0.019
7399	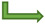	nervous system development				32	15.2	0.027
21785		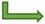	branchiomotor neuron axon guidance			3	0.06	0.02
48518	positive regulation of the biological process					77	49.5	0.012
31344	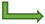	regulation of cell projection organization				17	4.36	0.003
120035		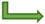	regulation of plasma membrane-bounded cell projection organization			17	4.29	0.003
51489			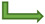	regulation of filopodium assembly		5	0.32	0.012
51491				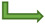	positive regulation of filopodium assembly	4	0.17	0.016
51239	regulation of multicellular organismal process					41	20.21	0.009
43536	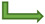	positive regulation of blood vessel endothelial cell migration				5	0.45	0.04
90050		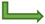	positive regulation of cell migration involved in sprouting angiogenesis			4	0.2	0.026
8299	isoprenoid biosynthetic process					4	0.24	0.043
48813	dendrite morphogenesis					5	0.46	0.042
22603	regulation of anatomical structure morphogenesis					19	6.88	0.033
10975	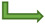	regulation of neuron projection development				14	2.84	0.002
50770		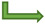	regulation of axonogenesis			8	1.13	0.013
50771			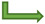	negative regulation of axonogenesis		5	0.48	0.048
34765	regulation of ion transmembrane transport					12	3.25	0.044
1904062	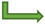	regulation of cation transmembrane transport				12	2.25	0.004
48583	regulation of response to stimulus					54	30.14	0.009
50920	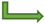	regulation of chemotaxis				9	1.78	0.037
32409	regulation of transporter activity					9	1.8	0.039
1655	urogenital system development					12	2.55	0.01
72001	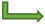	renal system development				10	2.29	0.044
44057	regulation of system process					14	3.57	0.013
65008	regulation of biological quality					56	30.03	0.003
42592	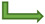	homeostatic process				29	12.43	0.013
48878		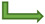	chemical homeostasis			23	8.89	0.019
51240	positive regulation of multicellular organismal process					25	10.62	0.033
10646	regulation of cell communication					45	25.74	0.048
9966	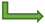	regulation of signal transduction				42	22.78	0.033
51179	localization					74	44.32	0.003
51234	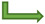	establishment of localization				60	33.77	0.004
6810		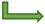	transport			57	32.4	0.009

**Table 6:** Descriptive statistics of total number born (with SD), number born alive (with SD), number of stillborn, and mummies per genotype of rs81300422 for sows ♀ and boars ♂ from a synthetic boar line and its crosses.

Line	Genotype	N	TNB	NBA	SB	Mummies
Synthetic boar line ♀	0/0	17,527	10.13 (3.10)	9.28 (3.10)	0.85	0.30
	0/1	3247	10.07 (3.05)	9.29 (3.02)	0.78	0.32
	1/1	115	10.03 (2.95)	9.46 (2.85)	0.57	0.39
Synthetic boar line ♂	0/0	25,500	10.08 (3.10)	9.23 (3.10)	0.85	0.33
	0/1	4333	9.95 (3.18)	9.12 (3.17)	0.83	0.37
	1/1	231	9.64 (3.01)	8.86 (2.94)	0.78	0.34
Crossbreed ^1^ ♀	0/0	32,015	16.56 (3.89)	15.39 (3.74)	1.17	0.39
	0/1	5290	16.52 (3.86)	15.30 (3.75)	1.21	0.35
	1/1	206	16.41 (4.08)	15.37 (3.84)	1.04	0.43
Crossbreed ^2^ ♂	0/0	54,824	16.00 (3.93)	15.06 (3.85)	0.94	0.36
	0/1	9714	16.02 (3.80)	15.05 (3.78)	0.97	0.36
	1/1	172	15.83 (4.08)	15.02 (3.92)	0.80	0.34

^1^ Synthetic boar line × (Large White × Landrace) ^2^ Synthetic boar line × (Landrace × Large White).

## Data Availability

Data of dataset **A** are contained within the article and supplementary materials. The data that support the findings of this study (combined in dataset **B**) are available from the National Engineering Research Center for Breeding Swine Industry and the Guangdong Provincial Key Lab of Agro-Animal Genomics and Molecular Breeding, College of Animal Science, South China Agricultural University (China); the Institute of Swine Science, Nanjing Agricultural University (China); Agrifood Research Finland, MTT, Biotechnology and Food Research (Finland); the TopigsNorsvin Research Centre, Beuningen (the Netherlands); and the NARIC Research Institute for Animal Breeding, Nutrition and Meat Science (Hungary), but restrictions apply to the availability of these data, which were used under license for the current study, and so are not publicly available. Data are however available from the authors upon reasonable request and with permission of: the National Engineering Research Center for Breeding Swine Industry and Guangdong Provincial Key Lab of Agro-Animal Genomics and Molecular Breeding, College of Animal Science, South China Agricultural University (China); the Institute of Swine Science, Nanjing Agricultural University (China); Agrifood Research Finland, MTT, Biotechnology and Food Research (Finland); the TopigsNorsvin Research Centre, Beuningen (the Netherlands); and the NARIC Research Institute for Animal Breeding, Nutrition and Meat Science (Hungary).

## Supplementary Materials

The following are available online at https://www.mdpi.com/article/10.3390/genes13101730/s1, Supplementary_Method: The description of not published GWAS [23], Table S1. SNPs associated with total number born in pigs with their location within pig genome (including chromosome - SSC, position and gene), alleles as well as breed in which the SNP was detected. Table S2. SNPs associated with number born alive in pigs with their location within pig genome (including chromosome - SSC, position and gene), alleles as well as breed in which the SNP was detected. Table S3. SNPs associated with number of stillborn in pigs with their location within pig genome (including chromosome - SSC, position and gene), alleles as well as breed in which the SNP was detected. Table S4. SNPs associated with litter birthweight in pigs with their location within pig genome (including chromosome, position and gene), alleles as well as breed in which the SNP was detected. Table S5. SNPs associated with corpus luteum number in pigs with their location within pig genome (including chromosome, position and gene), alleles as well as breed in which the SNP was detected.
