# Supplementary material for: Meta-Analysis of SNPs Determining Litter Traits in Pigs
Source: Genes (Basel). 2022 Sep 26;13(10):1730. doi: 10.3390/genes13101730 (PMC9602383; doi:10.3390/genes13101730)
Supplement: Supplementary file 1 [file genes-13-01730-s001.zip › Suppelementary_Method.pdf]

Unpublished data from:

Sell-Kubiak, E.; Duijvesteijn, N.; Lopes, M.S.; Janss, L.L.G.; Knol, E.F.; Bijma, P.; Mulder, H.A. Genome-wide association study reveals novel loci for litter size and its variability in a Large White pig population. BMC Genom. 2015, 16, 1049. https://doi.org/10.1186/s12864-015-2273-y.

Statistical analyses used for GWAS with G matrix

The single SNP genome wide association with the G-matrix (GRM) was performed based on the following model:

$$y = \mu + \text{SNP} + \text{line} + G + e,$$

where y is a deregressed EBV for the litter size or its variation; SNP is a fixed effect of the genetic marker; line is a fixed effect of a percentage of A-line in the animal (0% A-line or 12,5% A-line); and G is the vector of direct genetic effect of the animal obtained with the G-matrix. The G-matrix was used to account for the population stratification preset in this population of Large White. The G-matrix was calculated based on the following equation (VanRaden, 2008):

$$\mathbf{G} = \frac{\mathbf{ZZ'}}{\mathbf{2\sum p_i(1 - p_i)}},$$

where p_i is the frequency of the second allele at locus i, and the elements of **Z** were derived by subtracting doubled frequency of allele expressed as a difference of 0.5, i.e. $2(p_i – 0.5)$, from matrix **M** that specifies the 3 marker genotypes for each individual as −1, 0, or 1 (VanRaden, 2008).

Preliminary results GWAS

Results of the GWAS are based on single SNP association study with GRM. This was used as the test of data and initial expectation of the planned Multi-SNP GWAS with BayZ. After

accounting for population stratification by use of G-matrix none of the SNP remained significant (Figure 5 and 6). Thus next steps are:

1. Run single SNP association study with GRM, but use G-matrix after excluding most significant SNPs.
2. Run single SNP association study with A-matrix, subsequently perform the genomic control, were F-values are divided by the λ, and P-values are recalculated.

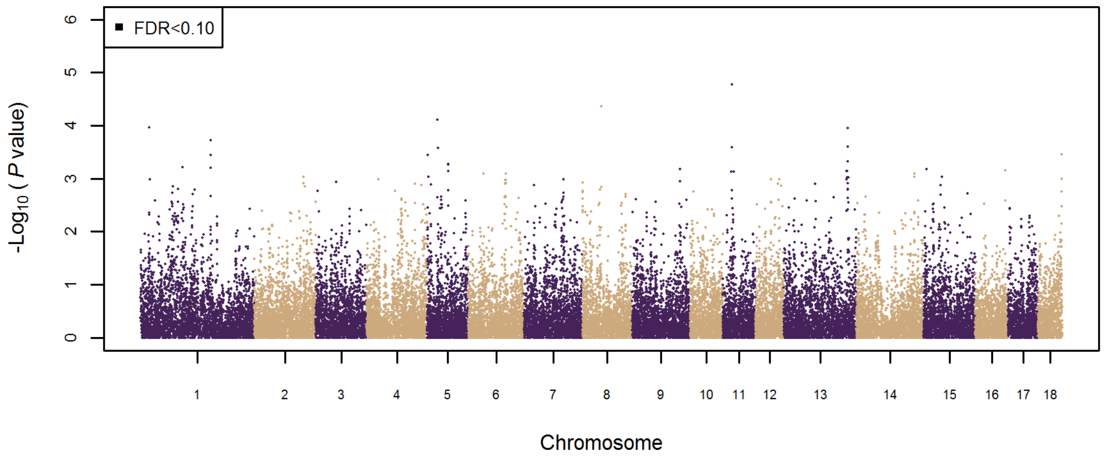

Figure S1. Single SNP GWAS with G-matrix on deregressed EBV of litter size.

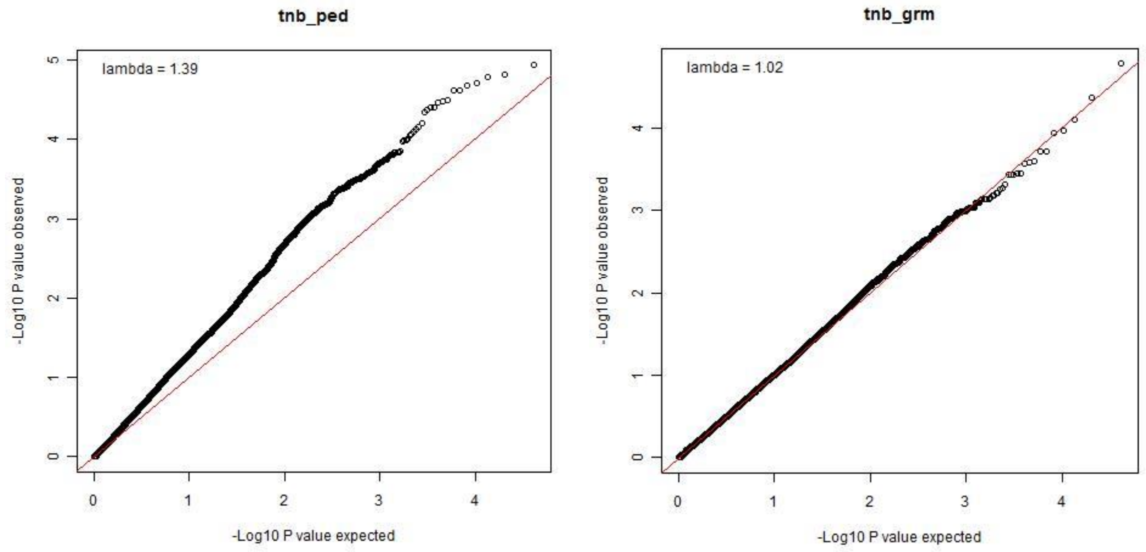

Figure S2. QQ plot of expected and observed –Log10 P-values of SNPs from the single SNP GWAS with pedigree (left figure) or with G-matrix (right figure) on deregressed EBV of litter size.
