# Supplementary material for: Meta-Analysis of SNPs Determining Litter Traits in Pigs
Source: Genes (Basel). 2022 Sep 26;13(10):1730. doi: 10.3390/genes13101730 (PMC9602383; doi:10.3390/genes13101730)
Supplement: Supplementary file 1 [file genes-13-01730-s001.zip › Supplementary information.pdf]

Supplementary information

Supplementary_Method: The description of not published GWAS performed by Sell-Kubiak et al. [23]

Supplementary_Tables: **Table S1.** SNPs associated with total number born in pigs with their location within pig genome (including chromosome - SSC, position and gene), alleles as well as breed in which the SNP was detected. **Table S2.** SNPs associated with number born alive in pigs with their location within pig genome (including chromosome - SSC, position and gene), alleles as well as breed in which the SNP was detected. **Table S3.** SNPs associated with number of stillborn in pigs with their location within pig genome (including chromosome - SSC, position and gene), alleles as well as breed in which the SNP was detected. **Table S4.** SNPs associated with litter birthweight in pigs with their location within pig genome (including chromosome, position and gene), alleles as well as breed in which the SNP was detected. **Table S5.** SNPs associated with corpus luteum number in pigs with their location within pig genome (including chromosome, position and gene), alleles as well as breed in which the SNP was detected. **Full references presented in Tables S1-S5**.
